# Type I IFN Promotes IL-10 Production from T Cells to Suppress Th17 Cells and Th17-Associated Autoimmune Inflammation

**DOI:** 10.1371/journal.pone.0028432

**Published:** 2011-12-06

**Authors:** Lixia Zhang, Shunzong Yuan, Genhong Cheng, Beichu Guo

**Affiliations:** 1 Department of Microbiology and Immunology, Hollings Cancer Center, Medical University of South Carolina (MUSC), Charleston, South Carolina, United States of America; 2 Department of Microbiology, Immunology and Molecular Genetics, University of California Los Angeles (UCLA), Los Angeles, California, United States of America; McMaster University, Canada

## Abstract

Whereas the immune system is essential for host defense against pathogen infection or endogenous danger signals, dysregulated innate and adaptive immune cells may facilitate harmful inflammatory or autoimmune responses. In the CNS, chronic inflammation plays an important role in the pathogenesis of neurodegenerative diseases such as multiple sclerosis (MS). Our previous study has demonstrated a critical role for the type I IFN induction and signaling pathways in constraining Th17-mediated experimental autoimmune encephalomyelitis (EAE), an animal model of human MS. However, it remains unknown if self-reactive Th17 cells can be reprogrammed to have less encephalitogenic activities or even have regulatory effects through modulation of innate pathways. In this study, we investigated the direct effects of type I IFN on Th17 cells. Our data show that IFNβ treatment of T cells cultured under Th17 polarizing conditions resulted in reduced production of IL-17, but increased production of IL-10. We also found that IFNβ induced IL-10 production by antigen specific T cells derived from immunized mice. Furthermore, IFNβ treatment could suppress the encephalitogenic activity of myelin-specific T cells, and ameliorate clinical symptoms of EAE in an adoptive transfer model. Together, results from this study suggest that IFNβ may induce antigen-specific T cells to produce IL-10, which in turn negatively regulate Th17-mediate inflammatory and autoimmune response.

## Introduction

Accumulating evidence indicates that chronic Inflammation is associated with a variety of human diseases. Therefore, constraining the inflammatory function of immune cells may provide a novel strategy to treat or control many chronic diseases, such as multiple sclerosis (MS) [1], [2], [3]. In response to pathogens, innate immune cells quickly upregulate pro-inflammatory cytokines that serve to initiate host defense against microbial invasion. However, excessive inflammation may cause tissue damage and activation of autoreactive T and B cells, which may have deleterious effects on a host. To prevent collateral damage and autoimmunity, hosts also develop a number of regulatory mechanisms, including generating Tregs and production of IL-10, to maintain homeostasis of the immune system. IL-10 is a potent anti-inflammatory cytokine with broad effects on both innate and adaptive immune systems [4], [5], [6], [7], [8], [9]. During bacterial or viral infection, IL-10 is produced by macrophages and DCs as a negative feedback mechanism to dampen uncontrolled production of inflammatory cytokines. In addition to innate cells, T cells, especially regulatory T cells, are able to produce IL-10 to inhibit the activation of antigen-specific cells and inflammatory response. Recently, studies from other and our groups indicate that type I IFN is able to exert its anti-inflammatory role through the induction of IL-10 and IL-27 from macrophages and DCs [9], [10], [11], [12].

When encountering specific antigens presented on APCs, naïve T cells differentiate into distinct subsets of effector cells. Depending upon cytokine milieu generated by macrophages and DCs, CD4 T cells can become different T helper subsets such as Th1, Th2, and Th17, or regulatory T cells such as Foxp3Treg and Tr1 cells [13], [14], [15], [16], [17], [18], [19], [20], [21], [22]. While Th1 cells are required for the clearance of intracellular pathogens, Th17 is involved in immune response against extracellular pathogens. On the other hand, Th17 cells have been shown to associate with pathogenesis of inflammatory autoimmune diseases, including MS and experimental autoimmune encephalomyelitis (EAE) [3], [23], [24], [25], [26], [27], [28]. Emerging evidence suggests that there is significant flexibility or plasticity among different Th subsets or between Th subsets and regulatory T cells [19], [29], [30], [31], [32], [33], [34]. MS and EAE are characterized by the infiltration of inflammatory cells, including macrophages and self-reactive T cells, into the central nervous system (CNS) that leads to neuron damage [2], [3], [8], [23], [35], [36], [37], [38], [39]. Recent studies suggest that Th17 cells, a novel subtype of CD4^+^ T helper cells, play an important role in the development of MS and EAE [3], [40], [41], [42], [43], [44]. However, experimental and clinical data indicate that CNS inflammation can result from over-activation of either Th1 or Th17, or both. Despite extensive studies, the cellular and molecular events triggering MS as well as regulatory mechanisms limiting the initiation and progression of CNS inflammation are still not well understood. To date, there are no curative treatments for MS.

Recent studies from other and our groups have demonstrated that IFNβ induction and signaling pathways play critical roles in suppressing Th17-associated autoimmune and inflammatory diseases including EAE [10], [11], [12], [43], [45]. The type I IFN, consisting of a single IFNβ and multiple IFNα members, is induced by TLR or cytoplasmic RNA and DNA sensors. IFNα and IFNβ bind to a common receptor, the type I IFN receptor (IFNAR), expressed on a wide variety of cell types, leading to induction of a large set of genes important for antiviral responses and other cellular functions. In addition to their antiviral functions, types I IFNs are capable of exerting immunomodulatory effects on both innate and adaptive immune cells. IFNα and IFNβ have been used to treat patients with cancer or autoimmune diseases, particularly multiple sclerosis [35], [45], [46], [47], [48], [49], [50], [51]. Our previous study shows that that type I IFN is required for LPS-induced IL-10 upregulation in macrophages [9]. Moreover, our recent studies revealed a novel immunoregulatory or immunosuppressive function of IFN induction pathway in innate and antigen-specific immune response. We found that mice lacking IFNα/β receptor (IFNAR) had enhanced development of Th17 cells *in vivo* and developed much more severe EAE than wild type mice. Experimental results from our laboratory and others have also provided critical links between IFNα/β and the induction of IL-27 from innate immune cells in the inhibition of Th17 cell differentiation. Recent studies suggest that IL-27-induced production of IL-10 may contribute to its immunosuppressive effects [52], [53], [54], [55], [56], [57].

Although our previous studies indicate that IFNβ is able to exert its anti-inflammatory role through macrophages and DCs, the direct effect of type I IFN on Th17 cell differentiation and plasticity remains less understood. As our previous studies show that IFNα/β can directly upregulate IL-10 expression in macrophages, we hypothesize that IFNβ may directly induce IL-10 production from T cells, consequently, inhibiting IL-17 production. Results from this study show that IL-10 expression was significantly upregulated in CD4 T cells upon IFN stimulation. Furthermore, IFN-treated encephalitogenic T cells generated less EAE *in vivo* in an adaptive transfer model. Data from this study support a novel mechanism in which IFNβ-induced IL-10 production may contribute to IFN-mediated inhibition of Th17-associated inflammation.

## Results

### Type I IFN directly acts on Th17 cells

**Figure 1:**
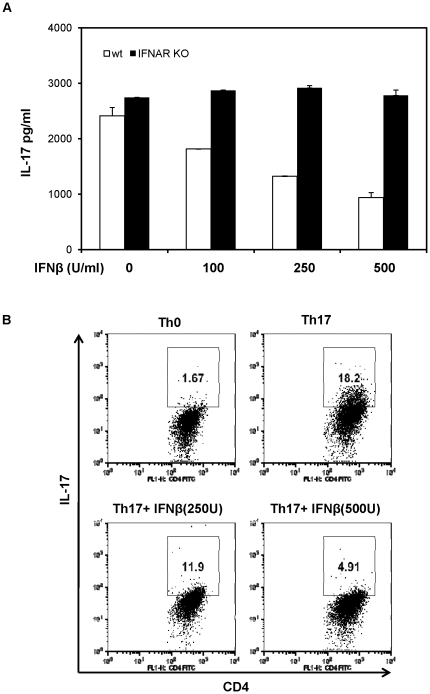
IFNβ directly acts on Th17 cells. (**A**) Naive CD4^+^ T cells from wt and IFNAR KO mice were purified by fluorescence-activated cell sorting, and stimulated with solid-phase anti-CD3 (5 µg/ml) and soluble anti-CD28 (2 µg/ml) under Th17 polarizing condition (TGFβ plus IL-6, and anti-IL-4/anti-IFNγ). IFNβ at different concentration was added at the beginning of culture. After 72 hours, IL-17 production in the culture supernatants was measured by ELISA. Results are reported as mean±SD of triplicate samples from one representative experiment. (**B**) Flow cytometry analysis of Th17 development in CD4 T cells cultured in the presence of IFNβ. Naive CD4 T cells were cultured in the Th17 condition in the presence of IFNβ (0, 250 U, 500 U/ml). After 72 hours, cells were stained for surface CD4 and intracellular IL-17. Plots were gated on CD4^+^ T cells. Data are representative of five independent experiments with similar results.

Our previous studies have demonstrated that type I IFN induction and signaling pathways in innate immune cells play an important role in limiting Th17-mediated autoimmune inflammation. This promoted us to determine if self-reactive Th17 cells can be modulated directly by IFN pathways. To test this hypothesis, we firstly examined the direct effect of type I IFN on Th17 differentiation and activity *in vitro*. Highly purified naïve CD4 T cells cultured under Th17 differentiation condition (IL-6 and TGFβ) were treated with IFNα or IFNβ, and then cytokine production and gene expression were analyzed. As shown in Figure 1A, while naïve T cells in the Th17 polarizing condition produced a significant amount of IL-17, adding exogenous IFNβ into the T cell culture led to decreased production of IL-17 in a dosage-dependent manner. In contrast to wt T cells, the production of IL-17 from IFNAR deficient T cultured under Th17 condition was not affected by type I IFN. Furthermore, intracellular cytokine staining reveled that IFNβ reduced the frequency of IL-17-positive T cells cultured in Th17-polarizing conditions (Fig. 1B).

**Figure 2:**
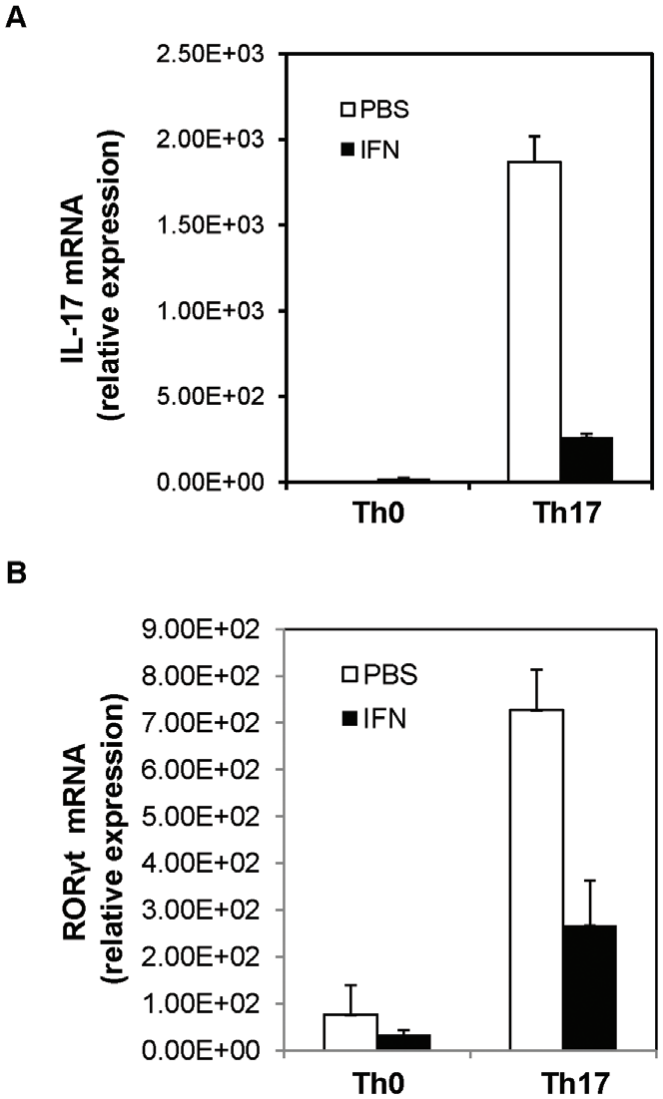
IFN-mediated inhibition of gene expression in Th17 cells. (**A**) The expression of IL-17A mRNA in Th17 cells treated with IFNβ. Naive T cells were cultured under Th17 condition in the presence of IFNβ (500 U/ml) for 3 days, the expression level of IL-17 mRNA was measured by Quantitative RT-PCR. Values are normalized to their average beta-actin values, and are presented as relative expression units. Error bars indicate ± SD among duplicate samples from one experiment. (**B**) Quantitative RT-PCR of the expression of mRNA encoding RORγt in Th17 cells treated with IFNβ. Values are normalized to their average beta-actin values and are presented as relative expression units. Error bars indicate ± SD among duplicate samples from one experiment. Data are representative of three independent experiments with similar results.

Differentiation of different CD4 T cell subsets is controlled by distinct transcriptional factors. Th17 differentiation program is orchestrated by the transcriptional factor RORγt, which itself is induced by TGFβ and IL-6. To determine the effect of IFNβ on the levels of gene expression in Th17 cells, the expression of mRNA encoding IL-17 and RORγ in Th17 cells treated with IFNβ was measured by Quantitative RT-PCR. Figure 2 shows that IFNβ could significantly inhibit RORγt expression in T cells stimulated with TGFβ and IL-6. Consequently, a direct treatment of naïve T cells cultured in Th17-polarizing condition with type I IFN led to reduced expression of IL-17 (Fig. 2).

### Type I IFN inhibits Th17 cells via both IL-10-dependent and -independent mechanisms

**Figure 3:**
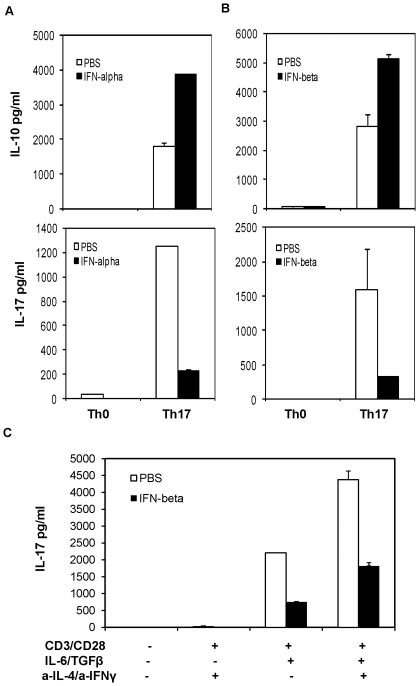
Type I IFN upregulates IL-10 production by Th17 cells. (**A**) and (**B**) Naïve CD4 T cells were cultured in Th17 culture conditions in the presence of purified IFNα or IFNβ (500 U/ml) for 72 hours. Production of IL-17 and IL-10 by CD4 T cells was determined by ELISA. (**C**) IL-10^−/−^ naive CD4 T cells were cultured in Th17 culture condition with IFNβ for 72 hours, the concentration of IL-17 in the supernatants was measured by ELISA. Results are reported as mean±SD of triplicate samples from one representative experiment. Data are representative of five (A, B) and three (C) experiments with similar results.

**Figure 4:**
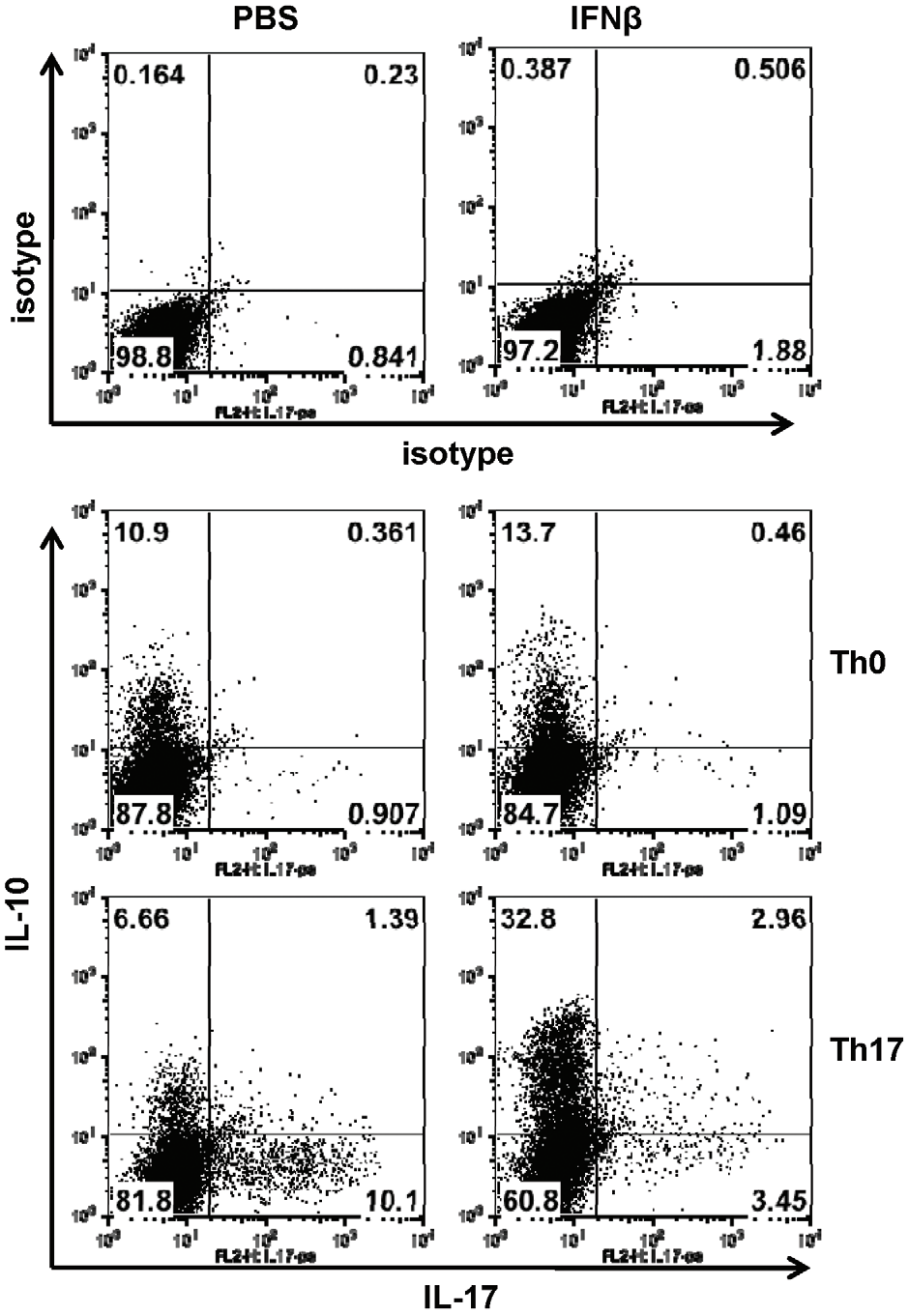
Upregulation of IL-10 expression in T cells by IFNβ. Naïve CD4 T cells were cultured in Th17 polarizing conditions as described above in the presence of IFNβ for 72 hours. Expression of IL-17 and IL-10 in CD4 T cells was determined by intracellular cytokine staining. Data are representative of five experiments with similar results.

Next, we would like to determine the mechanisms by which IFNβ directly inhibits IL-17 production from T cells. We hypothesized that IFNβ could potentially induce T cells under Th17 polarizing conditions to produce IL-10, which in turn limits IL-17 production. To test this, naïve CD4 T cells were cultured in Th17 culture conditions in the presence of recombinant IFNα or IFNβ for 72 hours, induction of IL-10 was measured by ELISA and intracellular cytokine staining. As shown in Figure 3A and 3B, IFNα and IFNβ induced IL-10 production but inhibited IL-17 production in Th17-polarized cells. Accordingly, FACS analysis revealed that IFNβ reduced the percentage of IL-17^+^ cells and increased IL-10^+^ T cell populations (Figure 4). We also found that treatment of IFNα/β increased percentage of IL-17 and IL-10 double positive T cells.

To determine if IFNβ-mediated inhibitory effects on Th17 cells is completely dependent on IL-10, we examined the effect of type I IFN on T cells from IL-10 KO mice in Th17 culture. While IL-10 deficient T cells produced more IL-17 compared with wt T cells, IFNβ could still exert certain degree of inhibition on Th17 cells, albeit in a less efficient manner (Fig. 3C). These results imply that IFN-induced IL-10 contributes to the negative regulatory function of IFNβ, but IL-10-independent mechanisms also exist in IFN-mediated inhibition of Th17 responses.

### Synergy between IFNα/β and IL-27 in the induction of IL-10

**Figure 5:**
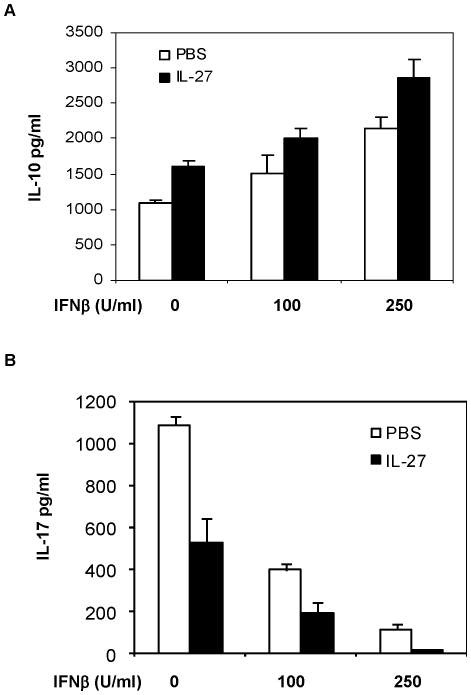
IL-27 and IFNβ synergistically induce IL-10 production by Th17 cells. (**A**) and (**B**) CD4 T cells were cultured under the Th17 conditions in the presence of IFNβ or IL-27 as indicated for 72 hours, the concentration of IL-17 or IL-10 in the culture supernatants was measured by ELISA. Results are reported as mean±SD of triplicate samples from one representative experiment. Data are representative of five experiments with similar results.

Our previous results show that IFNβ could induce IL-27 production from innate immune cells. Consistent with studies reported by other groups, we also found that IL-27 induced production of IL-10 in T-cells (data not shown). We further examined if IFNβ and IL-27 could cooperate in the modulation of Th17 cells. T cells in Th17 culture were treated with either IFNβ or IL-27 alone or together at different concentrations, the induction of IL-17 and IL-10 was measured by intracellular cytokine staining and ELISA. Interestingly, our data demonstrate that IFNβ and IL-27 acted in synergy to inhibit the production of IL-17, and to promote the induction of IL-10 in Th17 polarizing culture (Fig. 5A and 5B).

### Effects of IFNβ on Th17 proliferation and IL-10 production

**Figure 6:**
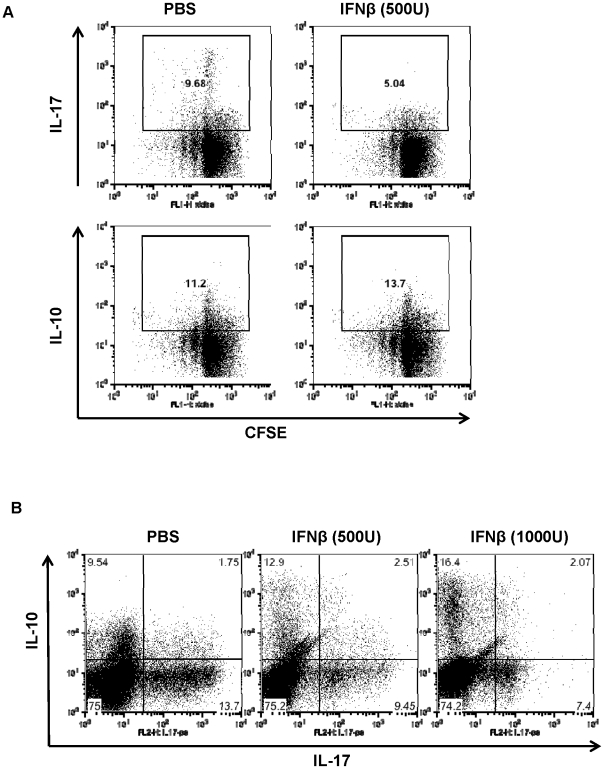
Mechanisms of IFN-mediated induction of IL-10 from T cells in Th17-polarizing conditions. (**A**). Proliferation of IL-10^+^ and IL-17^+^ CD4 T cells upon IFNβ treatment. Purified CD4 T cells were labeled with 5 µM of CFSE (carboxyfluorescein diacetate succinimidyl ester) and stimulated under the Th17 polarizing condition in the presence of IFNβ. After 3 days of culture, cells were stained for CD4, IL-10 and IL-17 and analyzed by FACS. Shown are representative CFSE profiles as well as intracellular IL-10 or IL-17 staining on gated CD4 cells. (**B**). Induction of IL-10 by IFNβ in fully differentiated Th17 cells. Naive CD4^+^ T cells were stimulated under the Th17-polarizing condition. After 3 days, T cells were re-cultured again for two more rounds under the same Th17 culture condition. In the third round of Th17 differentiation, T cells were treated with or without IFNβ. Expression of IL-17 and IL-10 was measured by intracellular cytokine staining and FACS analysis. Data are representative of three experiments with similar results.

The altered profile in IL-10/IL-17 production by type I IFN might be related to cytokine skewing or caused by preferentially promoting growth of IL-10 producing cells during T cell differentiation. To test these possibilities, we evaluated proliferation of IL-10 and IL-17 producing CD4 T cells by a CFSE labeling assay. T cells were labeled with the cytosolic dye CFSE, then cultured at Th17 condition in the presence of type I IFN. As shown in Figure 6A, both IL-10 and IL-17 producing T cells were actively dividing. Moreover, the rate of T cell proliferation in either IL-10^+^ or IL-17^+^ populations was generally comparable, although IL-10^+^ T cells divided slightly less than IL-17 positive T cells. These results indicate that IFN most likely promoted the expression of IL-10 from T cells, rather than selectively induced the expansion of IL-10 expressing T cells in Th17-polarizing conditions.

To further confirm if type I IFN could influence the fully differentiated Th17 cells, we cultured T cells for three rounds in Th17 polarizing conditions. In this experiment, naïve T cells were cultured in the presence of TGFβ and IL-6 for 3 days, then differentiated T cells were collected, washed and re-cultured under Th17 polarizing conditions for two more rounds. In the third round of Th17 culture, we treated differentiated Th17 cells with IFNβ. When these *in vitro* generated Th17 cells were cultured again in the Th17-polarizign condition (IL-6/TGFβ), IL-17 production was maintained. Consistent with our observations on naïve T cells, IFNβ treatment induced IL-10 production, but inhibited IL-17 production by T cells in the third round of Th17 polarizing culture (Figure 6B). Our data also show that IFNβ could still enhance the percentage of IL-17/IL-10 double positive T cells in such culture system. Although T cells after multiple rounds of differentiation culture may still contain mixed subpopulations, our data imply that IFNβ was able to modulate IL-17 and IL-10 production in well differentiated Th17 cells. These results also indicate that at least some of IL-10 could be produced by fully differentiating Th17 cells. However, further studies will be required to define the cell populations responsible for IFN-induced IL-10 production in T cells.

### Induction of IL-10 in antigen-specific T cells

**Figure 7:**
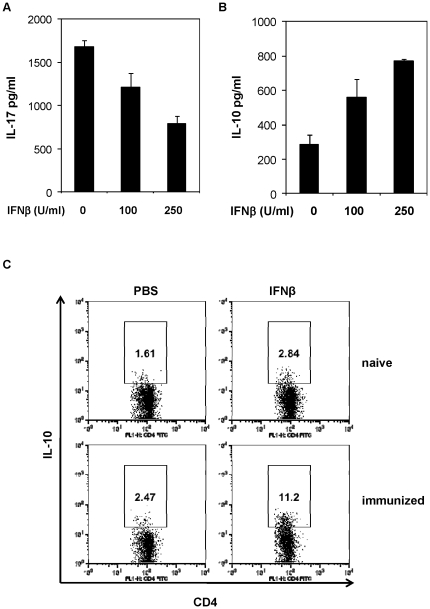
IFNβ promotes IL-10 production from antigen-specific T cells. (**A**) and (**B**) Wt mice were immunized with MOG peptide emulsified in CFA. On day 7 post immunization, total splenocytes were isolated and re-stimulated with MOG peptide ex vivo in the presence of IFNβ for 3 days, IL-17 and IL-10 production was measured by ELISA. Results are reported as mean±SD of triplicate samples from one representative experiment. (**C**) IFN-treated splenocytes as in (A) were stained for intracellular IL-10. Plots were gated on CD4^+^ T cells. Data are representative of three experiments with similar results.

Our results demonstrate that treatment of naïve T cells under Th17 polarizing condition with IFNβ could result in the increased expression and production of IL-10. We next investigated if IFNβ could induce IL-10 production from antigen specific Th17 cells. To test this, spleen cells isolated from MOG peptide-immunized mice were re-stimulated with the peptide *ex vivo* in the presence of IFNβ, and then the induction of IL-17 and IL-10 by T cells was measured by ELISA or FACS analysis. As reported previously, IFNβ inhibited IL-17 production from antigen-specific T cells (Figure 7A). Particularly, we found that IFNβ could induce IL-10 production from antigen-specific T cells generated from *in vivo* immune response (Figure 7B and 7C). Results from these studies suggest that IFNβ may induce antigen-specific T cells, possibly MOG-specific Th1 and Th17 cells, into IL-10-producing T cells.

### IFNβ decreases the encephalitogenic activity of antigen-specific Th17 cells and ameliorates symptoms of EAE

**Figure 8:**
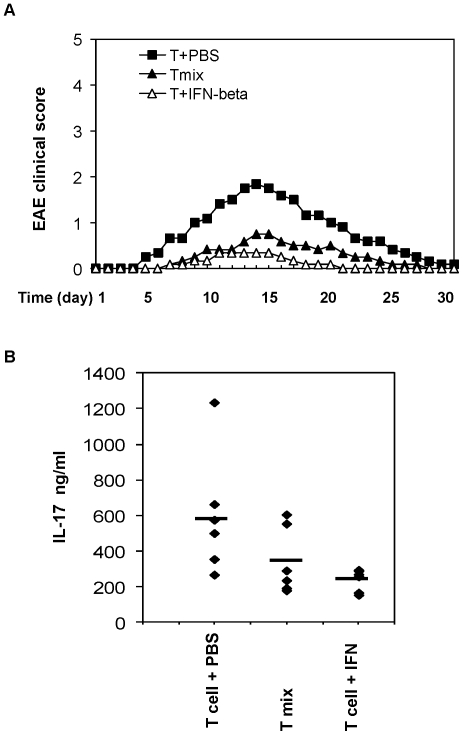
Encephalitogenic T cells treated with IFN lead to reduced EAE. (**A**) Wt mice were immunized with MOG peptide emulsified in CFA. On day 12 post immunization, total splenocytes were isolated and re-stimulated with MOG peptide ex vivo in the presence or absence of IFNβ for 3 days. Then CD4 T cells were purified and adoptively transferred into wt receipt mice. In addition, a mixture of antigen re-stimulated CD4 T cells containing both untreated and IFN-treated T cells (Tmix) were transferred into wt mice (5 mice per group). The mice were monitored daily for clinical sign of disease. (**B**) Total splenocytes were isolated from mice in (A) and re-stimulated with MOG peptide ex vivo, the IL-17 production was measured by ELISA. Results are reported as mean±SD of triplicate samples from one representative experiment. Data are representative of three experiments with similar results.

We next investigated effects of type I IFN on the pathogenic potential of self-reactive T cells. To test if IFNβ treatment can reduce the encephalitogenic activity of antigen-specific T cells, we utilized adoptive transfer experiments in which MOG-specific CD4 T cells were transferred into naïve wt mice. Spleen and lymph node cells isolated from immunized wt mice were re-stimulated *in vitro* with MOG peptide in the presence of IFNβ, then CD4 T cells reactive to MOG peptide were adoptively transferred into naïve mice to induce EAE. Three groups of cells were transferred into receipt mice: the first group was encephalitogenic T cells re-stimulated *in vitro* with the antigen only (T+PBS); in the second group, antigen stimulated T cells were also treated with IFNβ (T+IFN-beta); and in the third group, we co-transferred mixed T cells containing both T cells re-stimulated with MOG peptide alone and T cells re-stimulated with MOG peptide and IFNβ (Tmix). As shown in Figure 8A, encephalitogenic T cells induced EAE in recipient mice after adoptive transfer. However, transferring IFNβ-treated antigen specific T cells generated less severe EAE in recipient mice. Interestingly, the co-transfer of T cells treated with IFNβ delayed the onset and decreased the severity of EAE compared with transfer of untreated cells. Accordingly, lymphocytes isolated from recipient mice that received IFN-treated T cells produced less IL-17 proteins (Figure 8B). These results suggest that T cells treated with IFNβ may negatively regulate IL-17-producing T cells and autoimmunity *in vivo*.

## Discussion

In this study, we demonstrated that IFNβ treatment of naïve T cells in Th17 differentiation conditions resulted in decreased expression of IL-17 and RORγt. In contrast, treatment of IFNα/β led to increased number of both IL-10^+^ and IL-17^+^IL-10^+^ T cells, consequently enhanced IL-10 production in Th17 polarizing culture. We also found that IFNβ induced IL-10 production and reduced IL-17 production in antigen specific T cells derived from EAE mice. Furthermore, we found that treatment of myelin-specific T cells with IFNβ reduced their pathogenic function, and caused less severity of EAE in an adoptive transfer model. Results from this study imply that IFNβ may induce antigen-specific T cells to produce IL-10, thereby forming a negative feedback loop to regulate inflammatory and autoimmune response mediated by self-reactive T cells, probably including both Th17 and Th1 cells.

Our results suggest that type I IFN could suppress Th17-associated inflammation through multiple mechanisms, though further investigation is needed. Type I IFN may directly suppress the differentiation of Th17 cells, as evidenced by the inhibition of RORγt and IL-17 expression. During Th17 development, TGFβ and IL-6 induce naïve T cells to secrete IL-21, which functions as an autocrine factor to upregulate Th17 lineage-specific transcription factor RORγt and expression IL-23R [13], [14], [15], [16], [17], [18], [19], [20], [21], [22], [58]. It would be interesting to know if components of IFN signaling pathways can directly interact with molecules regulating Th17 differentiation. Another possible mechanism is that IFNβ may inhibit Th17 differentiation via induction of IL-10, which serves as a negative regulator for Th17 cells. IFNβ-mediated upregulation of IL-10 may suppress Th17 cells through inhibition of RORγt and IL-17 gene expression. Alternatively, IFN may promote the survival or expansion of IL-10 producing T cells. However, we observed similar level of dividing capacity in IL-10 producing T cells and IL-17 producing T cells when treated with IFNβ in CFSE labeling experiments, indicating that increased IL-10 producing T cells unlikely resulted from the outgrowth or survival of IL-10^+^ populations in our culture system. However, we can't absolutely exclude the possibility that IFN may selectively induce a particular IL-10^+^ T cell sub-population, especially during *in vivo* immune response. Therefore, further studies are required to define and track cell populations responsible for IFN-induced IL-10 production during immune response.

Previous work from our laboratory has shown that activation of type I IFN induction pathway constrains Th17 cells and EAE development. Our data suggest that IFNβ might indirectly inhibit Th17 cells via induction of IL-10 and IL-27 from macrophage and DCs. While induction of IL-27 may be one of important mechanisms for clinical benefits of IFNβ treatment, we hypothesize that the IFNβ-mediated IL-10 induction in T cells may represent an additional mechanism to inhibit Th17 cells and EAE. IL-27R deficient mice develop severe immunopathology in several infection and autoimmune models because of excessive inflammation, reminiscent the phenotypes of IL-10 and IFNAR deficient mice [52], [54], [55], [56], [58], [59]. In the context of autoimmunity, the overlapping EAE phenotypes of IL-27, IL-10, and IFNAR deficient mice suggest that these molecules are probably functionally linked in the regulation of Th17 development. Our data also show that IL-27 can induce IL-10 production from activated T cells. Whether Th17 cells can produce IL-10 is still controversial. A number of studies show that IL-6 plus TGFβ, IL-27 alone or with TGFβ, could induce T cells under Th17 conditions to upregulate IL-10 expression. However, some published studies suggest that no IL-10 is produced by Th17 cells [52], [53], [54], [55], [56], [57]. Nevertheless, our data suggest that Th17 cells could produce IL-10 in our experimental conditions. We also found either IFNβ or IL-27 could enhance IL-10 production from T cells. At present, it remains unclear regarding the relative contribution of IL-10 produced by different types of immune cells in normal and disease conditions.

Emerging evidence points to the heterogeneity and plasticity in Th17 cells. Our data suggest that IL-10 production from T cells, possibly Th17 cells and Tr1 cells, may act as a negative regulator to dampen inflammatory response mediated by antigen specific T cells or self-reactive T cells during autoimmune diseases. Although Th17 cells have been implicated in a number of autoimmune diseases, several recent reports suggest that Th17 cells have protective roles under certain conditions in inflammatory and autoimmune diseases. For example, McGeachy et al. reported that pathogenic Th17 cells cultured with TGFβ and IL-6 led to the generation of IL-10^+^ and IL-17^+^IL-10^+^ cells, which inhibited the pathogenic potential of Th17 cells and suppress the development of EAE in an adoptive transfer experiment of EAE [60]. The authors further suggest that IL-10 producing Th17 cells may have bystander suppressive effects to inhibit fully differentiated pathogenic Th17 populations, and the development of neuronal inflammation. O'Connor et al. also reported that IL-17 had a protective function in the development of T cell-mediated colitis [61]. It is possible that IFN-mediated IL-10 production from T cells could contribute to the inhibition of EAE development. We also noticed that IFNβ treatment led to an increase of IL-10^+^IL-17^+^ double positive cells among CD4 T cells under Th17 polarizing conditions. The induction of a population of IL-10/IL-17 double positive T cells suggests that IFNβ was able to elicit the expression and production of IL-10 from T cells that have differentiated into the Th17 cells. However, at present, it is not clear if IL-10/IL-17 double positive T cells are transit populations to IL-10 single positive cells, or a Th17 population transiently expressing IL-10. Nevertheless, our results show that IFNα/β could induce a small population of IL-10 and IL-17 double positive cells in T cells cultured under Th17 polarizing conditions. These results suggest that potential source of IL-10 in IFN-treated T cells may include both IL-10 single positive T cells and IL-10/IL-17 double positive T cells.

In summary, our data suggest that IFNβ-mediated IL-10 production from Th17 cells may contribute to the therapeutic efficacy of IFNβ. This result is also correlated with clinical observations that IFN treatment leads to increased production of IL-10 in MS patients. Furthermore, the co-transfer of T cells treated with IFNβ decreased the severity of EAE compared with transfer of untreated cells. These results imply that IFN-treated T cells may have regulatory effects *in vivo*. Despite efficacy of IFNβ in treating multiple sclerosis, the very short-half life and side effects limit its use. Our results suggest that *in vitro* induction of IL-10-producing T cells might provide an alternative strategy for the treatment of Th17-associated inflammatory diseases, such as EAE and MS. In addition to inducing IL-10 production, IFNβ is able to induce a number of signaling pathways and downstream genes. It would be important to elucidate the complexity and interaction of IFNβ-induced multiple genes and signaling pathways in regulating Th17 differentiation and autoimmune diseases.

## Materials and Methods

### Mice and reagents

All mice used are on a C57BL/6 genetic background. IFN Alpha R KO (IFNAR^−/−^) mice were from B&K Universal Limited (Grimston, Aldbrough, England). C57BL/6 wt mice and IL-10 KO mice were from the Jackson Lab. All animal experiments were approved by the Animal Research Committee (ARC) at UCLA (ARC#200901202), and the Institutional Animal Care & Use Committee (IACUC) at MUSC (AR#2998 and #3076), and were conducted in accordance with federal regulations as well as institutional guidelines and regulations on animal studies.

Murine IL-27, IL-6, TGFβ were purchased from R&D Systems (Minneapolis, MN); Murine IFNα and IFNβ proteins were from PBL Biomedical Laboratories (Piscataway, NJ); Anti-CD3, anti-CD28, anti-IFNγ, anti-IL-4, were from BD Biosciences (San Diego, CA). anti-IL-17 PE, anti-CD4 FITC, anti-IFNγ APC, anti-IL-10 APC and anti-Foxp3 APC were purchased from eBioscience (San Diego, California); MOG_35–55_ peptide were purchased from AnaSpec (San Jose, CA).

### EAE induction

For adoptive transfer-induced EAE, donor mice were immunized with MOG in CFA as described previously. Spleen cells and draining lymph node cells were isolated from mice 12 days after immunization and were re-stimulated with 20 µg/ml of MOG peptides *in vitro* for 72 hours in the presence or absence of IFNβ (500 U/ml). CD4 T cells were purified from ex vivo culture and washed extensively, and 3×10^7^ cells were transferred into each naive recipient mouse via *i.v.* injection (5 mice per group). On the same day and 2 days later each mouse received 200 ng of pertusis toxin via *i.v.* injection. The development of EAE in these receipt mice was monitored daily.

### Th17 cell culture

Single-cell suspensions were prepared from spleens of wt or different mutant mice. Naive CD4^+^ T cells were enriched from spleen mononuclear cells by magnetic cell sorting with a mouse CD4^+^ T cell isolation kit (Miltenyi Biotec), in some experiments as indicated, CD4 T cells were further purified by fluorescence-activated cell sorting. CD4 T cells were cultured in RPMI1640 (Gibco, BRL) supplemented with 10% FBS (HyClone), penicillin and streptomycin and 0.5 µM 2-mercaptoethanol. Polarization of naïve T cells into Th17 cells was achieved by culturing naive CD4^+^ T cells for three days with plate-bound antibody against CD3 (145-2C11, 5 µg/ml) plus soluble antibody against CD28 ( 2 µg/ml) in the presence of recombinant cytokine TGFβ1 (3 ng/ml), mouse IL-6 (20/ng ml). In Th0 condition, no cytokine was added. Where indicated, cultures were supplemented with anti–mouse IFN-γ (5 µg/ml), anti–mouse IL-4 (5 µg/ml), IFNα, IFNβ and IL-27 (5 ng/ml). In some experiments, T cells were cultured under Th17 condition for 3 days, then T cells were cultured for additional multiple rounds in the same Th17 polarizing condition with or without IFNβ.

### ELISA

IL-10, IL-17, IL-21, TNFα and IFNγ were detected in culture supernatants with ELISA sets or antibody pairs from BD Biosciences per the manufacturer's instructions.

### Flow cytometry

For intracellular cytokine staining, splenocytes or CD4 T cells were first re-stimulated for 4 hours with 50 ng/ml of phorbol 12-myristate 13-acetate (PMA) and 500 ng/ml of ionomycin in the presence of 5 µg/ml of brefeldin A (Sigma). Cells were surface-stained with fluorescein isothiocyanate-conjugated anti-CD4, then permeabilized with the Cytofix/Cytoperm Kit (BD Pharmingen or eBioscience) according to the manufacturer's protocol. Cells were stained intracellularly with phycoerythrin-conjugated anti–mouse IL-17 and allophycocyanin-conjugated anti–mouse IFN-γ, anti-mouse IL-10 or anti-mouse Foxp3. Samples were acquired on a FACSCalibur and data were analyzed with Flowjo software (TreeStar Inc).
